# Comparing performance of non–tree-based and tree-based association mapping methods

**DOI:** 10.1186/s12919-016-0063-4

**Published:** 2016-10-18

**Authors:** Katherine L. Thompson, David W. Fardo

**Affiliations:** 1 Department of Statistics, University of Kentucky, Lexington, KY 40536-0003 USA; 2 Department of Biostatistics, University of Kentucky College of Public Health, Lexington, KY 40536-0003 USA

## Abstract

A central goal in the biomedical and biological sciences is to link variation in quantitative traits to locations along the genome (single nucleotide polymorphisms). Sequencing technology has rapidly advanced in recent decades, along with the statistical methodology to analyze genetic data. Two classes of association mapping methods exist: those that account for the evolutionary relatedness among individuals, and those that ignore the evolutionary relationships among individuals. While the former methods more fully use implicit information in the data, the latter methods are more flexible in the types of data they can handle. This study presents a comparison of the 2 types of association mapping methods when they are applied to simulated data.

## Background

Linking variation in quantitative traits to locations along the genome (single nucleotide polymorphisms [SNPs]) is a central goal in the biomedical and biological sciences. The rapid growth of sequencing technology in recent decades has given researchers the ability to sequence genomes in a time-efficient and cost-efficient way. Development of statistical methods appropriate to search for associations among SNPs and quantitative traits (or phenotypes) has been quite active, often with complex data sets [1]. These complex scenarios include, but are not limited to, the case that the trait is simultaneously influenced by external covariates, multiple genes, or by gene–gene interactions. This complexity is exacerbated by the fact that many of the influences of SNPs on quantitative traits have only been through small effect sizes. Understanding these associations could prove useful in the disease diagnosis and/or treatment of human diseases, as well as in answering questions in evolutionary biology.

The search for SNPs associated with variation in a quantitative trait under study is often referred to as quantitative trait mapping (QTM). There are 2 specific goals of QTM: detection of SNPs associated with variation in quantitative trait(s) and localization of SNPs associated with variation in quantitative trait(s). In detection, the goal is to determine if any SNP in the region of the truly associated SNP is statistically significant. However, when analyzing hundreds of thousands of SNPs, the chance of detecting the exact causal locus is very small. As such, preferred methods will detect SNPs that are nearer to the causal SNP. This goal is referred to as *localization*.

Some association mapping methods (such as the classical *t*-test and those in McClurg et al. [2]) analyze genomic data efficiently and can detect strong associations among SNPs and a quantitative trait under study. In these methods, samples are grouped according to allele type at a single site or set of sites, and tests are performed to look for differences in trait value among such sites. However, these methods may miss weaker signals because they fail to use all available information, such as uneven evolutionary relatedness present in population-based samples of individuals.

**Fig. 1:**
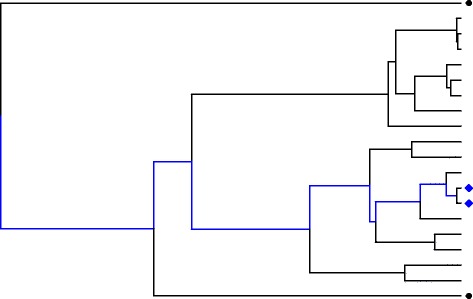
Example of the evolutionary history within a particular SNP represented by a phylogeny. In the phylogenetic tree, time moves from past (*left*) to present (*right*) across the tree. The tips of the tree represent observations from the present time. Suppose the SNP represented by this tree is associated with a trait. Then, a large covariance is expected among trait values from 2 observations (e.g., the *blue diamonds*) sharing a large portion of their evolutionary history (shown by the branches in *blue*). In contrast, the 2 observations denoted by *black circles* share a smaller portion of their evolutionary history, so that little covariance in the corresponding trait values is expected

Uneven relatedness among randomly sampled individuals can be attributed to the evolutionary history that exists within each SNP. Each SNP has an evolutionary history that can be represented by a bifurcating tree called a *phylogenetic tree*. For example, in the phylogenetic tree in Fig. 1, each tip represents a copy of the SNP shown. Time moves from past to present from left to right across the tree, and longer branch lengths represent longer times. For any 2 observations, the amount of shared evolutionary time is represented by the shared branches along the tree. Wherever 2 observations share a lineage (collection of branches), they share an evolutionary history. After a split in their lineages, the 2 observations evolve independently.

Consider the evolutionary history of a SNP associated with a trait. If 2 observations share a large portion of their evolutionary history (as is the case of the 2 blue diamonds in Fig. 1), their respective trait values are expected have a large correlation, whereas if 2 observations share little evolutionary history (as is the case of the 2 black circles in Fig. 1), their trait values are also expected to show little correlation. Thus, the evolutionary history of a SNP associated with a quantitative trait imposes a correlation structure on the trait values. Tree-based methods that use this correlation structure during analysis may have improved ability to detect weaker associations compared to methods that assume independence among observations.

Tree-based methods, such as those in Besenbacher et al. [3], Pan et al. [4], Thompson and Kubatko [5], Zhang et al. [6], and Zöllner and Pritchard [7], usually use the evolutionary history within each SNP, in the form of a phylogenetic tree, to gain information about the relationships among observations of the trait during analysis of genomic data. However, existing tree-based methods are also unable to consider complex, but biologically realistic data, such as external covariate data. In addition, by using more implicit information present in the data, tree-based methods also incur a computational cost. By using this implicit information, they may show improved performance compared to methods that are not tree based.

In this study, both a non–tree-based method and a tree-based method are used to directly compare performance of the 2 classes of methods. The analyzed data contains simulated phenotypes on 1943 unrelated individuals [8]. Five genes (*TNN*, *LEPR*, *GSN*, *TCIRG1*, and *FLT3*) were analyzed.

## Methods

The data analyzed was provided by the Genetics Analysis Workshop 19 [8]. Beagle was used to impute missing SNP data and to phase the genotypic data into haplotypes [9]. We analyzed SNPs in 5 genes and included 100,000 base pairs upstream or downstream from each gene (*TNN*, *LEPR*, *GSN*, *TCIRG1*, and *FLT3*). Locations of genes were determined using GeneCards Version 3 [10] and data was extracted using VCFtools Version 0.1.12b [11]. SNPs lacking 2 or more variants across samples were not analyzed.

The classical 2-sample *t*-test and the likelihood score statistic (LSS) approach [5] were studied to compare the performance of a non–tree-based method and a tree-based method. For the classical *t*-test, at each SNP, the chromosomal observations were partitioned according to SNP state (minor or major allele), and a pooled *t*-test was performed using these 2 groups. It should be noted that the number of observations is twice the number of individuals in the study.

For the LSS approach [5], at each SNP, a phylogenetic tree, denoted Θ, was estimated from the SNP data. Initial tree topology (tree shape) estimation was performed using the method from Mailund et al. [12], in which neighboring SNPs are used to estimate a phylogenetic tree at each SNP. To reduce computational expense, a broad-scale estimate of this tree is used. Specifically, the tree is considered to be a set of *k* clusters defined by the (*k* − 1) earliest splits in the tree. Each cluster of observations has a mean trait value (μ_i_). The covariance among 2 observations is proportional to the length of their shared lineages in the tree. The method assumes the trait values follow a multivariate normal distribution with a mean structure (**μ** = [μ_1_,μ_2_,…,μ_k_]), and covariance structure, V(Θ), defined by the estimated clustered tree. A constant variance, σ^2^, is assumed for all trait values. The LSS is based on the Bayesian information criterion and is defined to be the maximum penalized likelihood of the parameters given the data and the estimated tree. The likelihood is penalized by the number of parameters used in the calculation of the likelihood and the maximum is taken over the number of clusters (*k* = 2,3,…,*k*_*max*_). At the *i* ^*th*^ SNP, the score is defined to be:

$$ LS{S}_i=\underset{k}{ \max}\left\{2\  \ln L\left(\widehat{\boldsymbol{\mu}},{\widehat{\sigma}}^2\Big|\boldsymbol{y},V\left(\Theta \right),\Theta \right)-k \ln n\right\}. $$

Here, $\widehat{\boldsymbol{\mu}}$ and ${\widehat{\sigma}}^2$ are the maximum likelihood estimates of **μ** and σ^2^, respectively. The LSS treats each allele as an observation, so that the number of observations, *n*, is twice the number of individuals under study.

We have 2 goals to address—detection of the gene and localization of the causal loci—and we compare the performance of a tree-based method (the LSS) to a non–tree-based method (the classical *t*-test using chromosomes as observations as in Sasieni [13]) in terms of each goal. To address detection, permutation testing was performed. We created 100 permutation data sets by shuffling the trait values across genotypes, creating random trait–genotype pairs. The detection *p*-value was taken to be the proportion of permuted data sets producing test statistic values more extreme than the test statistic calculated using the observed data. To address localization, the average LSS and *t*-statistic values are shown at each SNP for comparison to the absolute true effect size at each SNP analyzed. The performance of the phylogenetic method (LSS) and the pooled 2-sample *t*-test were compared in the case of each goal.

## Results

Both the pooled *t*-test and the LSS (with a maximum of *k* = 15 clusters) were used to analyze systolic blood pressure using 5 genes from the 200 simulated phenotype data sets. All loci in all replicates chose far fewer than *k* = 15 clusters. We note that the alleles *t*-test does not account for phenotypic correlation between repeated measures across chromosomes. Four of these genes (*TNN*, *LEPR*, *FLT3*, and *GSN*) ranked in the top 9 genes in terms of total percentage variation in simulated systolic blood pressure (SBP). *TNN* and *LEPR* had effect sizes as large in magnitude as 10.89 and 11.99, respectively. We also analyzed genes accounting for a smaller percentage of the total variation in SBP, including *FLT3*, *TCIRG1*, and *GSN*, which had effect sizes as large in magnitude as 3.89, 3.38, and 0.76, respectively. Detection and localization performance was compared across the non–tree-based *t*-test and the tree-based LSS approach.

In terms of detection, both methods performed well in finding the signal present in *TNN* and *LEPR* (see rows 3 and 4 of Table 1). In the remaining 3 genes that had weaker signals (*FLT3*, *TCIRG1*, and *GSN*), much lower power was achieved by both methods, and neither method uniquely outperformed the other. In addition, 200 simulated Q1 trait data sets (data sets including a trait simulated without any direct genotype–phenotype association) were also analyzed to assess the type I error of each method. The LSS showed error rates lower than 0.05, whereas the *t*-test showed error rates above 0.05 in the case of all 5 genes (see rows 1 and 2 of Table 1). Although both methods showed similar power of detection, the *t*-test showed a type I error slightly larger than expected, whereas the LSS appeared somewhat conservative across the considered genes.

**Table 1:** Comparing power and type I error across methods using simulated data

Gene		*TNN* (97 SNPs)	*LEPR* (79 SNPs)	*FLT3* (87 SNPs)	*TCIRG1* (139 SNPs)	*GSN* (131 SNPs)
Type I error	LSS	0.010	0.045	0.020	0.015	0.020
	*t*-Statistic	0.075	0.100	0.085	0.100	0.100
Power	LSS	1.000	0.855	0.025	0.030	0.100
	*t*-Statistic	1.000	0.995	0.140	0.110	0.035

This table shows the power of detection for each of the 5 considered genes when considering 200 simulated Q1 (null) and 200 SBP phenotypes. The type I error of LSS appeared to be well controlled below 0.05 (row 1), whereas the *t*-statistic shows slightly inflated type I error rates (row 2). Both LSS and the *t*-test performed well when analyzing *TNN* and *LEPR* (rows 3 and 4). Both methods showed smaller power in the analysis of *FLT3*, *TCIRG1*, and *GSN* (rows 3 and 4). Neither method showed uniquely better performance across the 5 genes studied

**Fig. 2:**
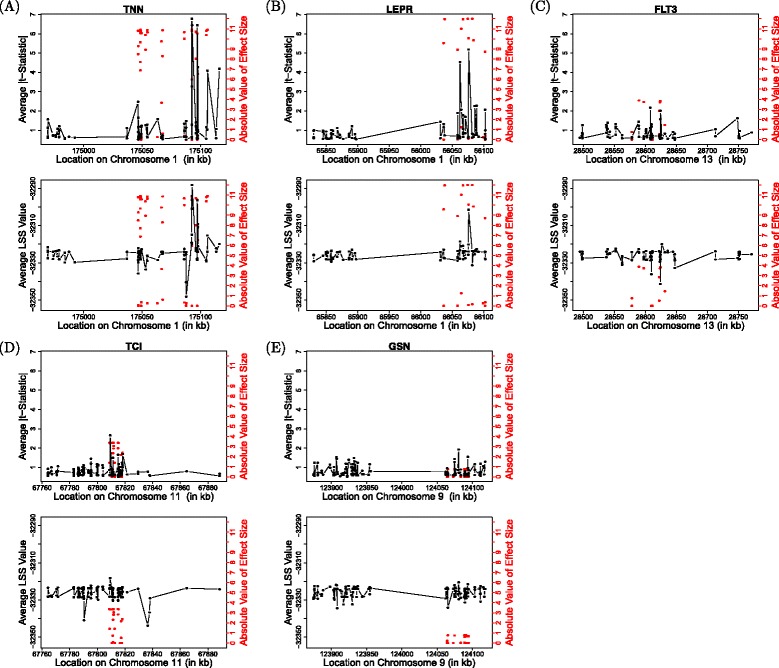
Example plot of each test statistic across the genes analyzed. Each upper plot in panels **a** to **e** shows the absolute value of the *t*-statistic against the location of each SNP along the chromosome. Each lower plot shows the average LSS value plotted against the base pair location of each SNP. Each *red dot* shows the true absolute value of the effect size at that respective locus. Both the *t*-test and LSS show similar precision in localizing the true absolute effects

Figure 2 shows the average statistic value at each SNP within each considered gene. Because the LSS should be larger in the case of an associated SNP, we consider the average LSS over the 200 replicate data sets at each SNP (shown in lower plots in Fig. 2). Similarly, for the *t*-test, we consider the average of the absolute value of the *t*-statistic over the 200 replicate data sets at each SNP (see upper plots in Fig. 2). The red dots in each plot show the true absolute value of the effect size at each locus (axis on right). As neither method shows a strong advantage in having higher values nearer to the truly causal variants, both the *t*-test and LSS appear to show similar ability to localize the true causal loci. However, LSS shows more similar values for nearby SNPs, while the *t*-test varies widely across each gene. This is expected because the *t*-test considers each SNP marginally, whereas LSS uses the estimated phylogenetic tree at each SNP, and, biologically, trees from neighboring SNPs along a chromosome are expected to be more similar than trees from SNPs farther apart. With respect to magnitude of effect size, the LSS showed higher power in detecting SNPs associated with simulated SBP than the 2-sample *t*-test in *GSN*, where the magnitudes of effect sizes within the gene were much smaller than in other cases. In the other cases with larger effects, the *t*-test outperformed the phylogenetic method. This may point to the reason that the methods had mixed performance when analyzing the considered genes.

## Discussion

In this study, genomic data were analyzed using both a classical non–tree-based approach (the 2-sample *t*-test) and a more recently proposed tree-based approach (the LSS). The results highlight the differences between the considered methods with respect to both the detection and the localization analyses. In the detection analysis, the methods showed similar power, but differed slightly in type I error rates. However, allelic tests can be biased [13], and this could be the root of the inflation in the type I error rate of the *t*-test. In a separate simulation study, we used an empirically derived critical value from the Q1 (null) trait data to make decisions about detection. In this case, the powers of detection were similar (within 2.5 %) of the results shown in Table 1 (data not shown). When considering localization, the LSS and the *t*-test had similar trends in estimating the location of the truly causal loci. In the case of a smaller effect size (*GSN*), the LSS outperformed the *t*-test, which may point to an advantage of using additional implicit information in genomic data sets to search for weaker genetic signals rather than excluding this information.

The study performed here was advantageous in that the simulated phenotypes were far more complex [8] than most previous simulated genetic data sets (e.g., see Besenbacher et al. [3], Pan et al. [4], Zhang et al. [6], and Zöllner and Pritchard [7]). However, the analyses were simplified in that they considered only the SNP data and phenotype of interest but ignored information about external variables (such as sex) present in the data. Although some existing non–tree-based methods are flexible enough to handle more complex biological scenarios such as the additional influence of external covariates on the trait under study, these methods fail to use all the information present in the data by ignoring the uneven evolutionary relatedness among the sampled individuals. Tree-based methods gain information about the covariance structure of the trait observations by considering the phylogenetic tree within each SNP, but incur a computational cost by doing so. In addition, existing tree-based methods are limited by an inelasticity to handle complex, but biologically realistic, data, such as data including external covariate information. As such, discussion and analyses here included SNP and phenotypic data to show an even comparison of the 2 classes of methods. However, as the phylogenetic method showed some promise in its ability to detect weaker genetic signals, developing tree-based techniques that are flexible enough to handle more complex data types could improve the success of association mapping using genomic data.

## Conclusions

Even though the rapid advancement of sequencing technology has produced large quantities of genomic data to analyze, improvements upon QTM methods continue to be developed. Non–tree-based methods exist to analyze complex data sets, but such methods tend to find large genetic effects. Currently, tree-based methods are able to analyze only basic genetic data sets, but more fully use implicit information in the genetic data to estimate the evolutionary relatedness among observations. These methods may improve upon QTM performance of non–tree-based methods, and could be extended so they are able to analyze more complex scenarios, such as inclusion of external covariates. Doing so would address a current limitation of tree-based association mapping methods and could improve the performance of methods in explaining variation in quantitative traits under study.
